# Prognostic Score Model for 30-Day Mortality in Patients with Acute Pulmonary Embolism Presenting to the Emergency Department

**DOI:** 10.3390/diagnostics16172850

**Published:** 2026-09-04

**Authors:** Shin Young Park, Incheol Park, Hyun Soo Chung, Yoo Seok Park, Soon Sung Kwon, Jinwoo Myung

**Affiliations:** 1 Department of Emergency Medicine, Severance Hospital, Yonsei University College of Medicine, 50-1 Yonsei-ro, Seodaemun-gu, Seoul 03722, Republic of Korea; chilli89@yuhs.ac (S.Y.P.); incheol@yuhs.ac (I.P.); hsc104@yuhs.ac (H.S.C.); pys0905@yuhs.ac (Y.S.P.); 2 Department of Laboratory Medicine, Gangnam Severance Hospital, Yonsei University College of Medicine, 50-1 Yonsei-ro, Seodaemun-gu, Seoul 03722, Republic of Korea

**Keywords:** pulmonary embolism, risk assessment, emergency department, prognosis, validation study

## Abstract

**Background/Objectives:** Early risk stratification is essential in pulmonary embolism (PE), but a simple tool integrating routinely available clinical and laboratory variables is lacking. We aimed to develop a simple score for predicting 30-day mortality in patients presenting to the emergency department (ED) with PE. **Methods:** We conducted a multicenter study in three hospitals in Korea. The score was derived at the largest hospital using least absolute shrinkage and selection operator logistic regression with bootstrap stability selection, and validated in the pooled cohort from the remaining two hospitals. Discrimination was assessed using the area under the receiver operating characteristic curve (AUROC) and compared with PESI and sPESI. Calibration was assessed using the Brier score and calibration parameters. **Results:** Among 2446 patients, 1753 were included in the derivation cohort and 693 in the validation cohort. The final score (3C score) assigned one point each for history of cancer, international normalized ratio ≥1.15, and C-reactive protein ≥50 mg/L. In the validation cohort, the AUROC was 0.767 (95% CI, 0.703–0.822) for 30-day mortality, compared with 0.748 for PESI and 0.725 for sPESI. 30-day mortality increased from 2.4% (score 0) to 34.4% (score 3). A score of 0 identified 42.7% of patients as low risk, with a negative predictive value of 97.6%. Calibration was acceptable (Brier score, 0.077; calibration slope, 1.098). **Conclusions:** The 3C score showed discrimination comparable to PESI and sPESI and identified a substantial subgroup with low risk. Its simplicity may facilitate ED risk assessment, although further validation is required before clinical implementation.

## 1. Introduction

Acute pulmonary embolism (PE) is a potentially fatal cardiovascular emergency, associated with substantial morbidity and early mortality across the spectrum of clinical presentations [1,2,3]. Despite improvements in diagnostic imaging and anticoagulation strategies, the short-term mortality following acute PE remains considerable, with reported 30-day all-cause mortality ranging from approximately 5% to 15% depending on the population studied [4]. Given this burden, accurate and timely risk stratification at the point of initial presentation—particularly in the emergency department (ED)—is essential to guide treatment intensity and disposition decisions.

Current guidelines from the European Society of Cardiology endorse the Pulmonary Embolism Severity Index (PESI) and its simplified version (sPESI) as the principal tools for prognostic stratification in hemodynamically stable patients with acute PE [4]. Both scores rely exclusively on clinical parameters available at the bedside—including age, vital signs, comorbidities, and oxygen saturation—and have been validated in multiple cohorts for identifying patients at low risk of early death [5,6,7]. However, despite their established utility, these scores have notable limitations. The original PESI encompasses 11 differentially weighted variables, making rapid bedside calculation impractical under time pressure. Moreover, the sPESI, while simpler, demonstrates relatively limited specificity in identifying truly high-risk patients, and neither score incorporates readily available laboratory data that may reflect the underlying physiological derangements of acute PE more precisely [8,9].

Beyond clinical scores, laboratory biomarkers have been investigated as prognostic adjuncts in acute PE, including cardiac troponin [10], which is already incorporated into current risk-stratification pathways [4]. Among biomarkers not captured by PESI or sPESI, several have shown independent prognostic value at ED presentation: elevated C-reactive protein (CRP) has been linked to thrombus propagation and end-organ injury [11]; reduced hemoglobin to impaired oxygen delivery [12]; prolonged prothrombin time (PT) to underlying coagulopathy or hepatic dysfunction [13,14]; and hypocalcemia to mid- and long-term mortality across multicenter cohorts [15,16].

Despite the accumulating evidence for these individual biomarkers, no validated scoring system has yet been developed that systematically integrates readily available laboratory parameters—such as coagulation indices and inflammatory markers—together with clinical variables in patients presenting to the ED with acute PE. Such a tool, if demonstrated to provide discriminative performance comparable to or better than existing clinical scores while retaining bedside simplicity, could meaningfully support risk stratification at the time of initial emergency evaluation.

We therefore aimed to develop a simple scoring system incorporating both clinical and routinely available laboratory parameters, and to validate this score in an independent multicenter cohort against PESI and sPESI.

## 2. Materials and Methods

### 2.1. Study Design

We conducted a retrospective derivation and validation study of a clinical prediction score model to predict 30-day mortality of patients who presented to the ED and were diagnosed with PE. Only patients with a confirmed diagnosis of PE by imaging in the ED between January 2010 and December 2025 were included. To develop and validate the score model, we conducted a multicenter cohort study involving three university-affiliated hospitals in Korea: Severance Hospital, Gangnam Severance Hospital, and Yongin Severance Hospital. The model was developed at Severance Hospital, a large tertiary referral hospital, and geographically validated using pooled cohorts from Gangnam Severance Hospital, a tertiary hospital, and Yongin Severance Hospital, a secondary hospital. Patients younger than 18 years, those without 30-day follow-up data, and those with any missing data in candidate variables were excluded. If the same patient had multiple eligible episodes, only the first episode was included in the analysis.

We collected candidate variables for the prediction model. Candidates were determined a priori considering their clinical associations with PE outcomes. Those variables were sex, age, systolic blood pressure (SBP), pulse rate (PR), respiratory rate (RR), body temperature (BT), oxygen saturation, mental status, calcium, hemoglobin, platelet count, creatinine, CRP, PT (expressed as international normalized ratio [INR]), history of chronic kidney disease, cancer, heart failure, diabetes, cardiac diseases other than heart failure, and chronic pulmonary diseases. All variables measured initially at ED presentation, before initiation of treatment, were collected.

This study was reviewed and approved by the Institutional Review Board of Yonsei University Health System (protocol code 3-2026-0012, approved on 13 March 2026), and was conducted in accordance with the principles of the Declaration of Helsinki. The requirement for informed consent was waived owing to the retrospective, de-identified nature of the study.

### 2.2. Outcomes

The primary outcome was 30-day mortality, defined as death within 30 days after the index ED presentation. The secondary outcome was in-hospital mortality, defined as death during the index hospitalization. Thirty-day mortality was retrospectively ascertained from the electronic medical records without requiring a prespecified inpatient or outpatient visit at 30 days. Survival status was considered ascertainable when either medical records were available beyond 30 days after the index visit or death within 30 days was documented in the available medical records.

### 2.3. Score Model Derivation

To facilitate bedside use in the ED, we sought to develop a score model containing a small number of variables that would be readily available during the initial ED evaluation.

First, we screened and selected variables to be included in the final score model. For variable selection, least absolute shrinkage and selection operator (LASSO) logistic regression was applied using all 20 prespecified candidate variables. Before variable selection using LASSO, continuous variables were dichotomized to facilitate construction of a simple bedside score. Binary cutoffs were determined jointly considering the cutoffs that maximized the Youden index in the derivation cohort and clinically established cutoffs used in existing score models or routine clinical practice.

The LASSO penalty parameter was selected using 5-fold cross-validation using binomial deviance as the optimization criterion. To favor a parsimonious model with similar cross-validated performance, the one-standard-error rule was used to determine the final penalty parameter for stability assessment.

To evaluate the robustness of variable selection, bootstrap stability analysis was performed in the derivation cohort. A total of 1000 bootstrap samples were generated by sampling patients with replacement, with each bootstrap sample having the same size as the original derivation cohort. In each bootstrap sample, LASSO logistic regression was tuned using internal 5-fold cross-validation, and the penalty parameter was selected according to the one-standard-error rule. A variable was considered selected when its LASSO regression coefficient was non-zero. Bootstrap selection frequency was defined as the proportion of the 1000 bootstrap samples in which each variable was selected.

Variables with high bootstrap selection frequencies were considered stable candidates. Only those selected in ≥95% of bootstrap samples were used for final model derivation. This threshold was chosen based on prior studies aimed at improving model generalizability and reducing overfitting [17,18]. Candidate score models were then constructed by sequentially adding candidate variables according to their bootstrap selection frequency. Each selected variable was assigned 1 point to preserve bedside simplicity. Then we assessed discrimination of each model using the area under the receiver operating characteristic curve (AUROC) in the derivation cohort. To balance discrimination and parsimony, we prespecified a minimum incremental AUROC of 0.01 for retaining an additional variable [19]. For each model, sensitivity, specificity, positive predictive value (PPV), negative predictive value (NPV), number of patients classified as low risk (score 0), and 30-day mortality in the low-risk group were also calculated.

### 2.4. Score Model Validation

After the final candidate score model was defined in the derivation cohort, its performance was evaluated in the validation cohort. Discrimination was evaluated using the AUROC. Sensitivity, specificity, PPV, NPV, number of patients classified as low risk (score 0), and the corresponding observed 30-day mortality were also evaluated.

Using the same validation cohort, we compared the performance of the derived score model with the existing score models, PESI and sPESI, in estimating 30-day mortality. We assessed model discrimination performance by AUROC.

### 2.5. Statistical Analysis

In the descriptive summaries, numbers (percentages) were reported for categorical variables. For continuous variables, means ± standard deviations (SDs) were reported. Model discrimination was assessed using AUROC. Ninety-five percent confidence intervals (CIs) for AUROC were estimated using patient-level percentile bootstrap resampling with 1000 repetitions. In each bootstrap replicate, patients were sampled with replacement from the corresponding analysis cohort, and the AUROC was recalculated. The 2.5th and 97.5th percentiles of the bootstrap AUROC distribution were used as the 95% CI.

For calibration assessment, separate logistic regression models were fitted in the derivation cohort for 30-day mortality and in-hospital mortality, with the total 3C score entered as the sole continuous predictor. The intercept and regression coefficient estimated for each outcome were applied without re-estimation to patients in the validation cohort to calculate individual predicted probabilities. For calibration plots, patients in the validation cohort were grouped according to their total 3C score, ranging from 0 to 3. Within each score category, observed mortality was calculated as the proportion of patients who experienced the outcome, and predicted mortality was calculated as the mean predicted probability. Predicted mortality was plotted against observed mortality, with the 45-degree line indicating perfect calibration. Overall calibration was assessed using the Brier score, calibration intercept, and calibration slope. Calibration intercept and slope were estimated in the validation cohort by fitting a logistic recalibration model with the observed outcome as the dependent variable and the logit of the predicted probability as the only predictor. Internal calibration of the final 3C score in the derivation cohort was assessed using bootstrap optimism correction with 1000 resamples. The optimism-corrected Brier score, calibration intercept, and calibration slope were calculated.

Statistical analyses were performed using R software, version 4.3.2 (R Foundation for Statistical Computing, Vienna, Austria).

## 3. Results

### 3.1. Baseline Characteristics of the Study Cohort

**Figure 1:**
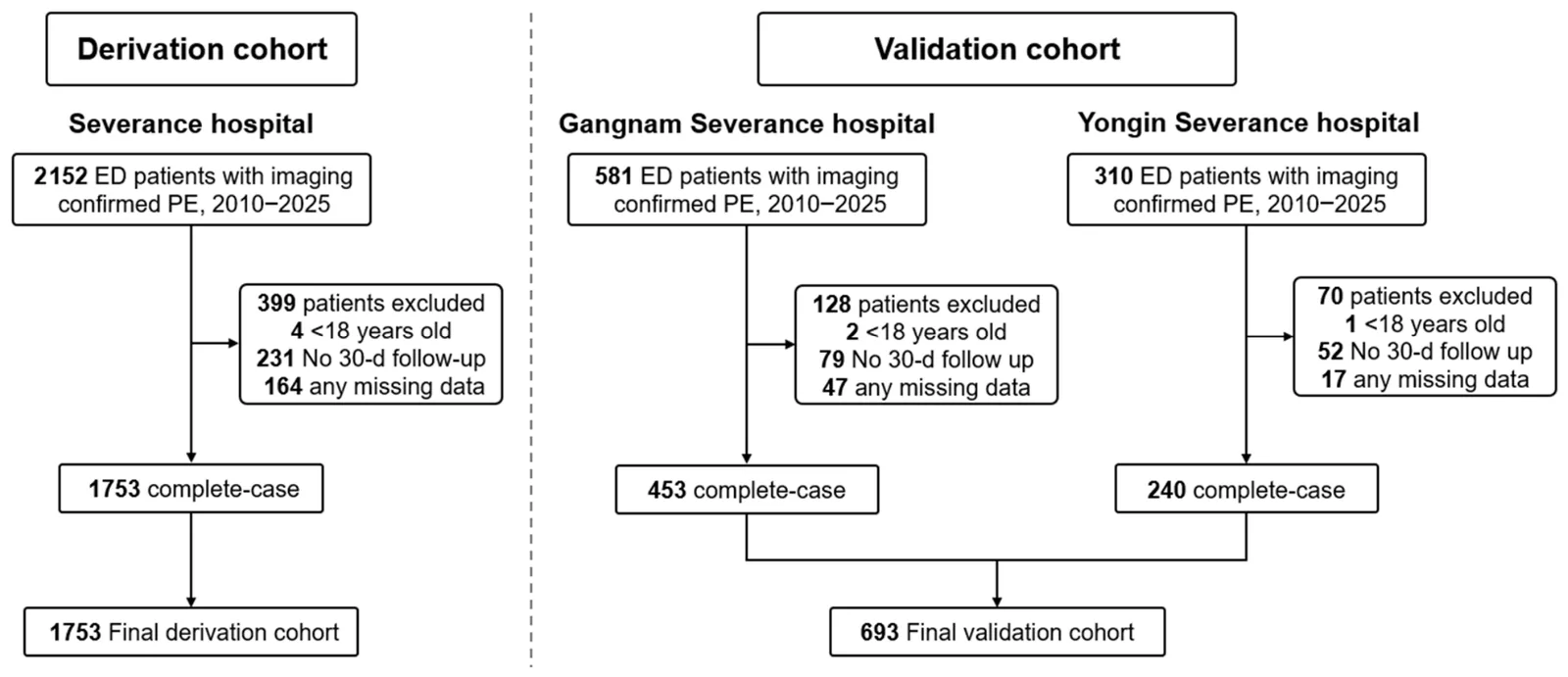
Flowchart for participant inclusion and exclusion.

During the study period, 3043 patients presented to the EDs of participating hospitals and were diagnosed with PE. Among those, patients younger than 18 years old (n = 7), those without 30-day follow-up data (n = 362) and those with any missing data (n = 228) were excluded. Subsequently, 2446 patients were included in model derivation and validation, including 1753 patients in the derivation cohort (mean age, 65.61 ± 14.85 years; 841 [48.0%] male) and 693 patients in the validation cohort (mean age, 66.49 ± 17.98 years; 294 [42.4%] male). Detailed numbers of patients and the selection process were described in Figure 1. The 30-day mortality was 17.7% in the derivation cohort and 9.4% in the validation cohort. The in-hospital mortality was 16.7% in the derivation cohort and 10.2% in the validation cohort.

Table 1 indicates patient characteristics in the derivation and validation cohorts. The derivation cohort showed lower SBP, higher PR and RR, and lower oxygen saturation. Hemoglobin level was lower in the derivation cohort, and CRP and INR were higher in the derivation cohort. While other comorbidities were similar between cohorts, the proportion of patients with a history of cancer was higher in the derivation cohort.

### 3.2. Model Derivation

Before model derivation, continuous variables were dichotomized: age ≥ 45 years, SBP < 100 mmHg, PR ≥ 110/min, RR ≥ 20/min, BT < 36.0 °C, oxygen saturation < 90%, calcium ≤ 8.1 mg/dL, hemoglobin < 11 g/dL, platelet count < 150 × 10^9^/L, creatinine ≥ 1.1 mg/dL, CRP ≥ 50 mg/L, and INR ≥ 1.15.

Then LASSO with bootstrap was used to select variables to be used in the score model. As a result of LASSO regression, eight variables had bootstrap selection frequencies of at least 95%: history of cancer, INR ≥ 1.15, CRP ≥ 50 mg/L, history of diabetes, RR ≥ 20/min, platelet count < 150 × 10^9^/L, BT < 36.0 °C and PR ≥ 110/min. Selection frequencies of all variables were described in Supplementary Table S1. Then we developed models with the number of variables from two to eight, in order of proportion selected in the LASSO regression with 1000 bootstraps (Supplementary Table S2). Addition of CRP ≥ 50 mg/L to the two-variable score model (history of cancer and INR ≥ 1.15) increased the AUROC from 0.744 to 0.760. Addition of history of diabetes further increased the AUROC by 0.007. The three-variable model was selected according to the prespecified criteria: history of cancer, INR ≥ 1.15, and CRP ≥ 50 mg/L (Table 2). The model showed moderate discrimination performance with high NPV for rule-out patients with low risk. The model classified 26.0% of patients in the derivation cohort as low risk, with a 30-day mortality of 2.9% in the low-risk group. This model was named the 3C score (history of Cancer, INR [Coagulation], and CRP). After bootstrap correction for optimism, the Brier score, calibration intercept, and calibration slope for 30-day mortality in the derivation cohort were 0.127, 0.009, and 1.002, respectively.

### 3.3. Model Validation

**Figure 2:**
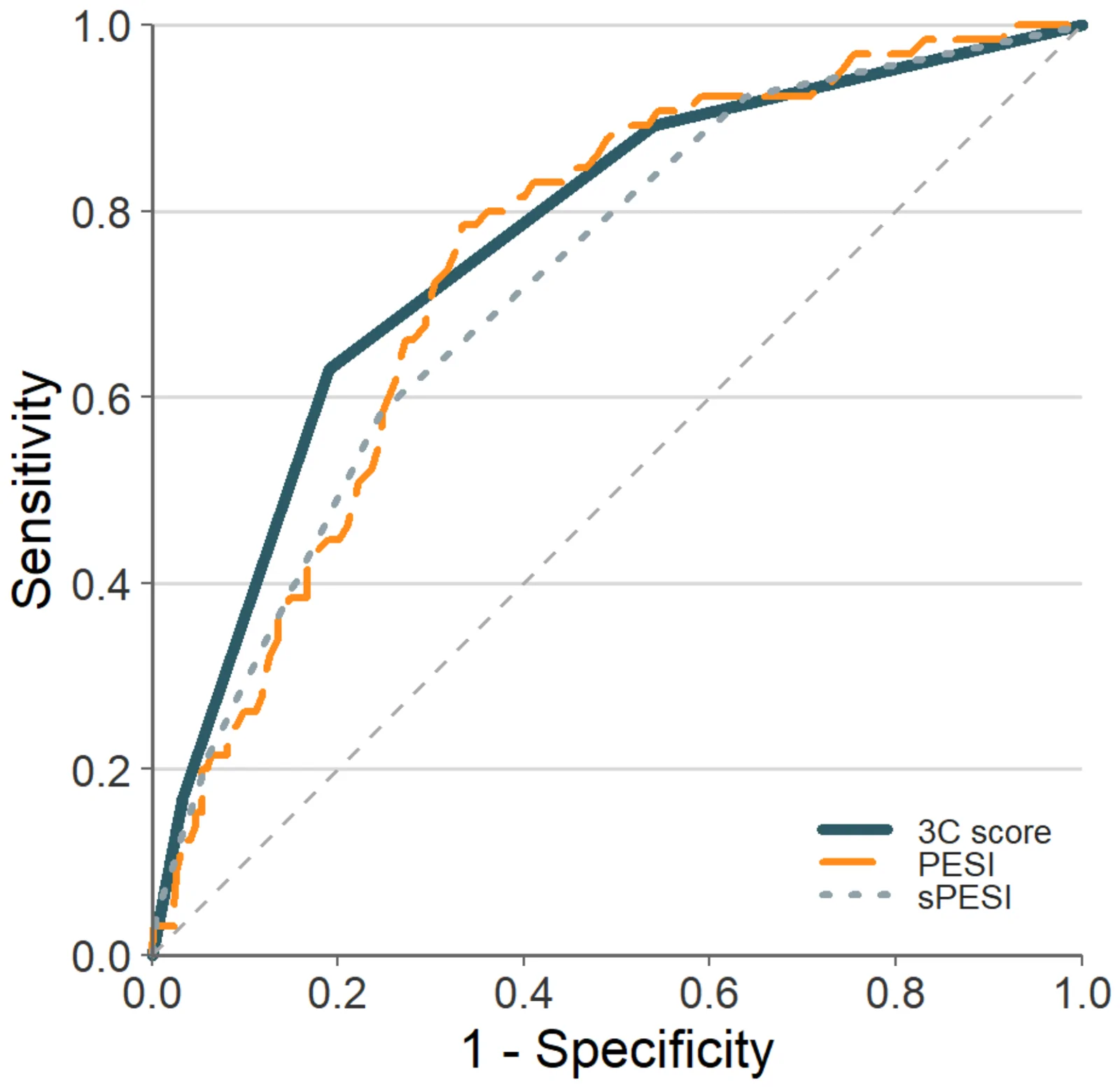
The receiver operating characteristic curves of the 3C score, PESI, and simplified PESI for predicting 30-day mortality in the validation cohort.

The performance of the 3C score was evaluated in the validation cohort. The AUROC was 0.767 (95% CI 0.703–0.822), which was comparable to PESI (AUROC 0.748; 95% CI 0.688–0.802) and sPESI (AUROC 0.725; 95% CI 0.666–0.781) (Figure 2).

Four risk groups could be defined with corresponding mortality rates determined. The 3C score effectively discriminated patients according to the risk of 30-day mortality: 2.4% for score 0, 7.2% for score 1, 23.3% for score 2, and 34.4% for score 3.

Performance of the 3C score for predicting 30-day mortality was described in Table 3. When a cutoff of <1 was applied to identify patients at low risk of 30-day mortality, the 3C score showed high sensitivity (89.2%) and NPV (97.6%). The score classified 42.7% of the validation cohort as low risk, with a 30-day mortality of 2.4%. The sensitivity and NPV were comparable to those of PESI (89.2% and 97.7%, respectively) and sPESI (92.3% and 97.8%, respectively). The proportions of patients classified as low risk were 43.3% with PESI and 33.5% with sPESI (Supplementary Table S3).

The performance for predicting in-hospital mortality was also evaluated in the validation cohort. The AUROC was 0.790 (95% CI 0.734–0.842), which was comparable to PESI (AUROC 0.740; 95% CI 0.681–0.795) and sPESI (AUROC 0.726; 95% CI 0.669–0.778). The 3C score also effectively discriminated patients according to the risk of in-hospital mortality, and could effectively identify patients with a low risk of in-hospital mortality (Supplementary Table S4).

Calibration of the 3C score was assessed in the validation cohort by comparing observed mortality with predicted mortality. For 30-day mortality, the model showed mild overall overprediction, with observed and predicted mortality of 9.4% and 11.4%, respectively. Overprediction was more apparent in the highest score category, whereas observed and predicted risks were closely aligned at score 2 (Supplementary Figure S1A). Overall calibration was acceptable, with a Brier score of 0.077, calibration intercept of −0.073, and calibration slope of 1.098.

For in-hospital mortality, overall observed and predicted mortality were closely aligned at 10.2% and 10.6%, respectively. The model slightly overpredicted mortality in the lower score categories, underpredicted mortality at score 2, and showed close agreement at score 3 (Supplementary Figure S1B). Calibration was acceptable, with a Brier score of 0.080, calibration intercept of 0.370, and calibration slope of 1.232.

## 4. Discussion

In this multicenter derivation and validation study of patients presenting to the ED with acute PE, three readily available variables at ED presentation—history of cancer, INR ≥ 1.15, and CRP ≥ 50 mg/L—were combined into a simple score, the 3C score, that showed consistent discrimination for 30-day and in-hospital mortality across the derivation cohort and two separate hospital-based validation cohorts. In the validation cohort, the three-item score showed numerically higher discrimination than both PESI (AUROC, 0.767 vs. 0.748) and sPESI (AUROC, 0.767 vs. 0.725), while requiring only three dichotomous items compared with 11 weighted variables for PESI and six variables for sPESI. The score effectively classified patients as low risk, among whom the observed 30-day mortality was low (2.4%) and the NPV was 97.6%, suggesting potential utility for identifying a low-risk subgroup at ED presentation. The 3C score can be calculated during the initial ED evaluation, following confirmation of the diagnosis of PE and once the initial laboratory results are available, allowing early prognostic assessment.

Cancer is common among patients with PE [20] and is a well-established independent risk factor for mortality [21,22,23]. Both PESI and sPESI incorporate cancer as a predictor of mortality [5,7]. Consistently, cancer was common in our cohort, and a history of cancer was a stable predictor of mortality. INR is not included in established PE mortality risk scores and has received relatively limited attention as a prognostic marker in PE; nevertheless, it was one of the most stable predictors of mortality in our cohort. This finding is consistent with two previous studies reporting an association between elevated INR and mortality in patients with PE [13,14]. Although CRP is also not incorporated into established PE prognostic scores, previous studies have demonstrated its prognostic relevance in PE. Elevated CRP has been associated with right ventricular dysfunction [24,25], early mortality [11], and PE-related 30-day mortality [24], although its independent association with all-cause mortality has not been consistent.

The clinical utility of the 3C score lies primarily in its simplicity. It comprises three binary variables obtained from routine history taking and standard laboratory tests and is calculated by simple addition, providing a simple estimate of early mortality risk in time-constrained ED settings. Although the requirement for laboratory results precludes calculation at the moment of triage, the incorporation of laboratory variables distinguishes the 3C score from entirely clinical scores such as PESI and sPESI and may capture different aspects of early mortality risk.

In the validation cohort, patients with a 3C score of 0 had a 30-day mortality of 2.4%. Similarly, low-risk patients identified by PESI and sPESI had 30-day mortality rates of 2.3% and 2.2%, respectively. These findings suggest that low-risk classification by any of these scores should not be used alone to support early discharge or outpatient treatment. Accordingly, the 3C score is intended to complement rather than replace established PE risk-stratification strategies. It may inform the intensity of monitoring and the urgency of further assessment, while decisions regarding treatment and disposition should continue to incorporate hemodynamic status, cardiac biomarkers, right ventricular dysfunction, and bleeding risk.

This study has several strengths. First, the score was developed using a relatively large multicenter cohort of 2446 patients with imaging-confirmed PE, providing an adequate number of outcome events for model development and evaluation. Second, the model underwent geographic external validation at two additional hospitals rather than being assessed solely by internal validation. Importantly, the derivation and validation cohorts differed substantially in baseline characteristics, including cancer prevalence, clinical severity, laboratory findings, and mortality rates. Despite these differences, the 3C score maintained consistent discrimination and acceptable calibration in the validation cohort, supporting its robustness across clinically heterogeneous populations. Finally, PESI and sPESI were evaluated in the same validation cohort, allowing direct comparison under identical clinical conditions.

This study has several limitations. First, its retrospective design may have introduced selection bias and misclassification of clinical variables and outcomes. Second, patients without ascertainable 30-day survival status or with missing values for any candidate predictor were excluded, which may have affected the representativeness of the study population. Although patients with unavailable 30-day follow-up did not exhibit a lower baseline risk profile than those included in the analysis (Supplementary Table S5), selection bias cannot be completely excluded and may have affected model performance. Third, the study spanned a 15-year period (2010–2025); although the diagnostic approach to PE has remained largely stable over this interval, treatment practices evolved substantially. Fourth, comprehensive guideline-based PE risk classification could not be performed because cardiac troponin and measures of right ventricular dysfunction were unavailable in a substantial proportion of patients. In addition, anatomical PE distribution and concomitant DVT status were not systematically collected. Therefore, the incremental value of the 3C score when combined with guideline-based risk-stratification pathways remains to be determined. Finally, differences in referral patterns and patient characteristics across the participating hospitals may have contributed to the difference in mortality between the derivation and validation cohorts, while the inclusion of only university-affiliated hospitals may limit the generalizability of our findings to other healthcare settings.

The 3C score effectively identifies patients at low risk of 30-day mortality among patients presenting to the ED with PE using three variables readily available at ED presentation. Its simplicity makes it a practical candidate for bedside risk stratification. By providing a rapid and low-burden estimate of early mortality risk, the 3C score may complement established risk-stratification pathways during the initial ED assessment of patients with PE. Prospective validation in more diverse clinical settings is warranted before the score is used to guide management decisions.

## Figures and Tables

**Table 1:** Baseline characteristics of the derivation and validation cohorts.

Characteristic	Derivation (n = 1753)	Validation (n = 693)
Demographics		
Age, years	65.61 ± 14.85	66.49 ± 17.98
Male sex	841 (48.0)	294 (42.4)
Vital signs and mental status		
Systolic blood pressure, mmHg	123.86 ± 26.33	130.02 ± 26.16
Pulse rate,/min	99.98 ± 21.32	95.55 ± 21.31
Respiratory rate,/min	19.14 ± 4.32	18.48 ± 3.43
Body temperature, °C	36.91 ± 0.81	36.82 ± 0.79
Oxygen saturation, %	95.20 ± 4.94	95.78 ± 5.54
Altered mental status	93 (5.3)	31 (4.5)
Laboratory values		
Hemoglobin, g/dL	11.52 ± 2.38	12.10 ± 2.53
Platelet count, 10^9^/L	234.48 ± 132.13	233.74 ± 101.52
Creatinine, mg/dL	0.94 ± 0.76	0.91 ± 0.76
Calcium, mg/dL	8.51 ± 0.78	8.67 ± 0.70
C-reactive protein, mg/L	73.70 ± 81.72	60.86 ± 72.06
Prothrombin time (INR)	1.17 ± 0.49	1.10 ± 0.32
History of comorbidities		
Chronic kidney disease	65 (3.7)	28 (4.0)
Cancer	933 (53.2)	169 (24.4)
Heart failure	35 (2.0)	21 (3.0)
Diabetes	340 (19.4)	119 (17.2)
Cardiac diseases other than heart failure	220 (12.5)	92 (13.3)
Chronic pulmonary disease	131 (7.5)	38 (5.5)
Outcomes		
30-day mortality	311 (17.7)	65 (9.4)
In-hospital mortality	293 (16.7)	71 (10.2)

INR, international normalized ratio.

**Table 2:** Components and point assignments of the 3C (Cancer, Coagulation, C-reactive protein) score for prediction of 30-day mortality of patients with pulmonary embolism.

Variables	Points
History of cancer	
No	0
Yes	1
Prothrombin time (INR)	
<1.15	0
≥1.15	1
C-reactive protein (mg/L)	
<50	0
≥50	1

Total score ranges from 0 to 3. INR, international normalized ratio.

**Table 3:** Performance of the 3C score for predicting 30-day mortality in the validation cohort.

Cutoff	≥Cutoff		<Cutoff		Sensitivity (%)	Specificity (%)	PPV (%)	NPV (%)
	Patients, n (%)	Mortality (%)	Patients, n (%)	Mortality (%)				
1	397 (57.3)	14.6	296 (42.7)	2.4	89.2	46.0	14.6	97.6
2	161 (23.2)	25.5	532 (76.8)	4.5	63.1	80.9	25.5	95.5
3	32 (4.6)	34.4	661 (95.4)	8.2	16.9	96.7	34.4	91.8

PPV, positive predictive value; NPV, negative predictive value.

## Data Availability

The data presented in this study are available on request from the corresponding author due to institutional data-sharing restrictions related to patient privacy.

## Supplementary Materials

The following supporting information can be downloaded at: https://www.mdpi.com/article/10.3390/diagnostics16172850/s1, Table S1: Bootstrap selection frequencies of all candidate variables; Table S2: Performance of candidate scoring models in the derivation cohort; Table S3: Performance of the 3C score, PESI, and sPESI for identifying patients at low risk of 30-day mortality in the validation cohort; Table S4: Performance of the 3C score for predicting in-hospital mortality; Table S5: Comparison of baseline characteristics between patients with available and unavailable 30-day follow-up; Figure S1: Calibration plots for 30-day and in-hospital mortality.

## References

[1] Goldhaber S.Z. (1998). Pulmonary embolism. N. Engl. J. Med..

[2] Bělohlávek J., Dytrych V., Linhart A. (2013). Pulmonary embolism, part I: Epidemiology, risk factors and risk stratification, pathophysiology, clinical presentation, diagnosis and nonthrombotic pulmonary embolism. Exp. Clin. Cardiol..

[3] Martin K.A., Molsberry R., Cuttica M.J., Desai K.R., Schimmel D.R., Khan S.S. (2020). Time Trends in Pulmonary Embolism Mortality Rates in the United States, 1999 to 2018. J. Am. Heart Assoc..

[4] Konstantinides S.V., Meyer G., Becattini C., Bueno H., Geersing G.J., Harjola V.P., Huisman M.V., Humbert M., Jennings C.S., Jiménez D. (2020). 2019 ESC Guidelines for the diagnosis and management of acute pulmonary embolism developed in collaboration with the European Respiratory Society (ERS). Eur. Heart J..

[5] Aujesky D., Obrosky D.S., Stone R.A., Auble T.E., Perrier A., Cornuz J., Roy P.M., Fine M.J. (2005). Derivation and validation of a prognostic model for pulmonary embolism. Am. J. Respir. Crit. Care Med..

[6] Bumroongkit C., Limsukon A., Liwsrisakun C., Deesomchok A., Pothirat C., Theerakittikul T., Trongtrakul K., Tajarernmuang P., Niyatiwatchanchai N., Inchai J. (2023). Validation of the Pulmonary Embolism Severity Index Risk Classification and the 2019 European Society of Cardiology Risk Stratification in the Southeast Asian Population with Acute Pulmonary Embolism. J. Atheroscler. Thromb..

[7] Jiménez D., Aujesky D., Moores L., Gómez V., Lobo J.L., Uresandi F., Otero R., Monreal M., Muriel A., Yusen R.D. (2010). Simplification of the pulmonary embolism severity index for prognostication in patients with acute symptomatic pulmonary embolism. Arch. Intern. Med..

[8] Palas M., Silva B.V., Jorge C., Almeida A.G., Pinto F.J., Caldeira D. (2022). The accuracy of Hestia and simplified PESI to predict the prognosis in pulmonary embolism: Systematic review with meta-analysis. TH Open.

[9] Moini C., Lay P., Jochmans S., Azandjo F., El Karroumi N., Bouilland A.-L., Hafiani E.M. (2026). Risk stratification in pulmonary embolism: The expanding role of biomarkers. Biomedicines.

[10] Elmewafy A.T., Waller J., Alaguraja P., Desor K., Antoun I. (2025). The Prognostic Value of Troponin in Acute Pulmonary Embolism: A Systematic Review and Meta-Analysis. Catheter. Cardiovasc. Interv..

[11] Büyükşirin M., Anar C., Polat G., Karadeniz G. (2021). Can the Level of CRP in Acute Pulmonary Embolism Determine Early Mortality?. Turk. Thorac. J..

[12] Obradovic S., Dzudovic B., Subotic B., Salinger S., Matijasevic J., Benic M., Kovacevic T., Kovacevic-Kuzmanovic A., Mitevska I., Miloradovic V. (2023). Association of Blood Leukocytes and Hemoglobin with Hospital Mortality in Acute Pulmonary Embolism. J. Clin. Med..

[13] Kırış T., Yazıcı S., Durmuş G., Çanga Y., Karaca M., Nazlı C., Dogan A. (2018). The relation between international normalized ratio and mortality in acute pulmonary embolism: A retrospective study. J. Clin. Lab. Anal..

[14] Wong C.C.Y., Ng A.C.C., Lau J.K., Chow V., Chen V., Ng A.C.T., Yong A.S.C., Sindone A.P., Marwick T.H., Kritharides L. (2016). High mortality in patients presenting with acute pulmonary embolism and elevated INR not on anticoagulant therapy. Thromb. Haemost..

[15] Wang X., Xiang Y., Zhang T., Yang Y., Sun X., Shi J. (2020). Association between serum calcium and prognosis in patients with acute pulmonary embolism and the optimization of pulmonary embolism severity index. Respir. Res..

[16] Yang Y.Q., Wang X., Zhang Y.J., Chen Y.F., Wang L., Hu X.W., Niu L., Pu H.M., Zhang X., Zhang Z. (2022). Prognosis assessment model based on low serum calcium in patients with acute pulmonary thromboembolism. Respirology.

[17] Xie R., Herder C., Sha S., Peng L., Brenner H., Schöttker B. (2024). Novel type 2 diabetes prediction score based on traditional risk factors and circulating metabolites: Model derivation and validation in two large cohort studies. eClinicalMedicine.

[18] Huang Z., Klaric L., Krasauskaite J., Khalid W., Strachan M.W.J., Wilson J.F., Price J.F. (2023). Combining serum metabolomic profiles with traditional risk factors improves 10-year cardiovascular risk prediction in people with type 2 diabetes. Eur. J. Prev. Cardiol..

[19] Martens F.K., Tonk E.C., Kers J.G., Janssens A.C. (2016). Small improvement in the area under the receiver operating characteristic curve indicated small changes in predicted risks. J. Clin. Epidemiol..

[20] Timp J.F., Braekkan S.K., Versteeg H.H., Cannegieter S.C. (2013). Epidemiology of cancer-associated venous thrombosis. Blood.

[21] den Exter P.L., Gómez V., Jiménez D., Trujillo-Santos J., Muriel A., Huisman M.V., Monreal M. (2013). A clinical prognostic model for the identification of low-risk patients with acute symptomatic pulmonary embolism and active cancer. Chest.

[22] Park D.Y., An S., Kashoor I., Ezegwu O., Gupta S. (2022). In-hospital prognosis of malignancy-related pulmonary embolism: An analysis of the national inpatient sample 2016–2018. J. Thromb. Thrombolysis.

[23] Sedhom R., Beshai R., Moussa P., Megaly M., Mohsen A., Abramov D., Stoletniy L., Elgendy I.Y. (2024). Outcomes with Malignancy-Associated High-Risk Pulmonary Embolism: A Nationwide Analysis. Mayo Clin. Proc..

[24] Abul Y., Karakurt S., Ozben B., Toprak A., Celikel T. (2011). C-reactive protein in acute pulmonary embolism. J. Investig. Med..

[25] Najarro M., Rodríguez C., Morillo R., Jara-Palomares L., Vinson D.R., Muriel A., Álvarez-Mon M., Yusen R.D., Bikdeli B., Jimenez D. (2024). C-reactive Protein and Risk of Right Ventricular Dysfunction and Mortality in Patients with Acute Symptomatic Pulmonary Embolism. Arch. Bronconeumol..

