# Supplementary material for: Prognostic Score Model for 30-Day Mortality in Patients with Acute Pulmonary Embolism Presenting to the Emergency Department
Source: Diagnostics (Basel). 2026 Sep 4;16(17):2850. doi: 10.3390/diagnostics16172850 (PMC13564852; doi:10.3390/diagnostics16172850)
Supplement: Supplementary file 1 [file diagnostics-16-02850-s001.zip › diagnostics-4519447-supplementary.pdf]

Supplementary Figure S1. Observed versus predicted probabilities of 30-day mortality (A) and in-hospital mortality (B) in the validation cohort (perfect calibration; dashed line).

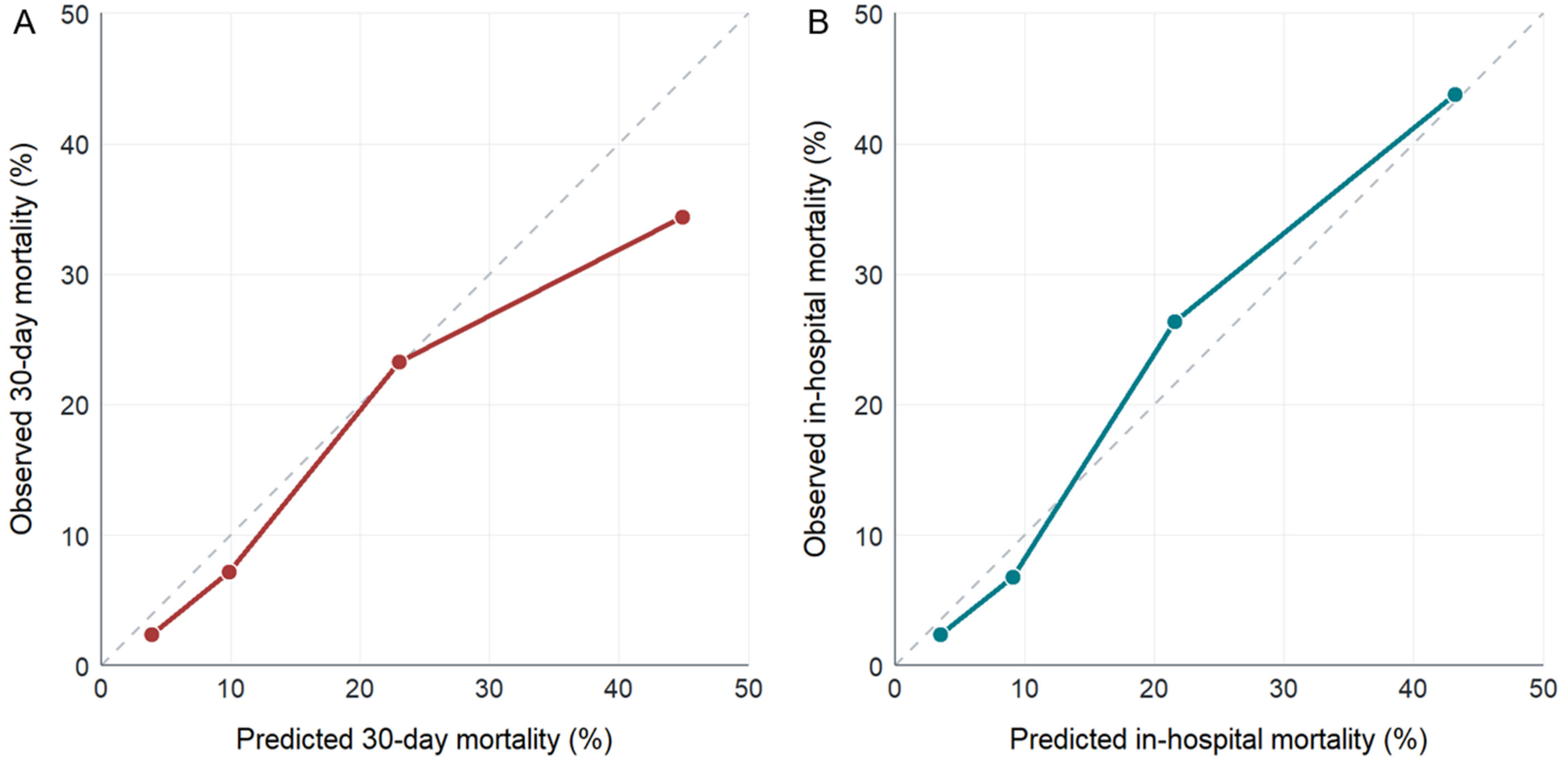

Supplementary Table S1. Bootstrap selection frequencies of candidate variables across 1,000 bootstrap samples using least absolute shrinkage and selection operator logistic regression.

Rank	Variable	Selection frequency (%)
1	History of cancer	100
2	Prothrombin time (International normalized ratio [INR] ≥1.15)	100
3	C-reactive protein ≥50 mg/L	99.8
4	History of diabetes	98.2
5	Respiratory rate ≥20/min	98.1
6	Platelet count <150x10^9/L	97.9
7	Body temperature <36℃	97.2
8	Pulse rate ≥110/min	96.7
9	Oxygen saturation <90%	92.0
10	Age ≥45y	84.3
11	Systolic blood pressure <100 mmHg	75.1
12	Calcium ≤8.1 mg/dL	67.4
13	History of cardiac disease other than heart failure	62.3
14	History of chronic pulmonary disease	56.5
15	Altered mental status	46.6
16	Male sex	35.9
17	Creatinine ≥1.1 mg/dL	32.7
18	Hemoglobin <11 g/dL	27.8
19	History of chronic kidney disease	26.6
20	History of heart failure	24.6

Supplementary Table S2. Performance of candidate scoring models in the derivation cohort.

Model	AUC	Sensitivity (%)	Specificity (%)	PPV (%)	NPV (%)	Low-risk patients	Low-risk mortality (%)
Cancer + PT(INR) ≥1.15	0.744	91.3	42.1	25.4	95.7	634 (36.2)	4.3
+ CRP ≥50 mg/L	0.760	95.8	30.7	23.0	97.1	455 (26.0)	2.9
+ Diabetes	0.767	96.8	26.6	22.1	97.5	394 (22.5)	2.5
+ RR ≥20/min	0.772	97.7	15.1	19.9	96.9	225 (12.8)	3.1
+ Platelet count <150x10^9/L	0.775	97.7	12.8	19.5	96.3	191 (10.9)	3.7
+ BT <36°C	0.777	98.1	12.0	19.4	96.6	179 (10.2)	3.4
+ PR ≥110/min	0.782	99.0	9.6	19.1	97.9	142 (8.1)	2.1

For all candidate models, a score of 0 was classified as low risk and a score of ≥1 as testing positive.

AUC, area under curve; PPV, positive predictive value; NPV, negative predictive value; PT, prothrombin time; INR, international normalized ratio; CRP, C-reactive protein; RR, respiratory rate; PR, pulse rate; BT, body temperature.

Supplementary Table S3. Performance of the 3C score, PESI, and sPESI for identifying patients at low risk of 30-day mortality in the validation cohort.

Model	Patients, n (%)	Mortality (%)	Sensitivity (%)	Specificity (%)	PPV (%)	NPV (%)
3C = 0	296 (42.7)	2.4	89.2	46.0	14.6	97.6
PESI ≤85	300 (43.3)	2.3	89.2	46.7	14.8	97.7
sPESI = 0	232 (33.5)	2.2	92.3	36.1	13.0	97.8

PPV, positive predictive value; NPV, negative predictive value

Supplementary Table S4. Performance of the 3C score for predicting in-hospital mortality in the validation cohort.

Cutoff	≥ Cutoff		< Cutoff		Sensitivity (%)	Specificity (%)	PPV (%)	NPV (%)
	Patients, n (%)	Mortality (%)	Patients, n (%)	Mortality (%)				
1	397 (57.3)	16.1	296 (42.7)	2.4	90.1	46.5	16.1	97.6
2	161 (23.2)	29.8	532 (76.8)	4.3	67.6	81.8	29.8	95.7
3	32 (4.6)	43.8	661 (95.4)	8.6	19.7	97.1	43.8	91.4

PPV, positive predictive value; NPV, negative predictive value.

Supplementary Table S5. Comparison of baseline characteristics between patients with available and unavailable 30-day follow-up.

Characteristic	30-day survival status available (n=2,446)	30-day survival status unavailable (n=310)
**Demographics**		
Age, years	65.86 ± 15.80	72.16 ± 14.50
Male sex	1,135 (46.4%)	119 (38.4)
**Vital signs and mental status**		
Systolic blood pressure, mmHg	125.60 ± 26.42	126.42 ± 27.49
Pulse rate, /min	98.73 ± 21.40	100.64 ± 20.84
Respiratory rate, /min	18.95 ± 4.09	19.22 ± 4.09
Body temperature, °C	36.88 ± 0.81	36.92 ± 0.83
Oxygen saturation, %	95.37 ± 5.13	95.86 ± 4.55
Altered mental status	124 (5.1)	38 (12.3)
**Laboratory values**		
Hemoglobin, g/dL	11.69 ± 2.44	11.38 ± 2.20
Platelet count, 10^9/L	234.27 ± 124.21	230.44 ± 108.28
Creatinine, mg/dL	0.93 ± 0.76	0.92 ± 0.63
Calcium, mg/dL	8.56 ± 0.76	8.48 ± 0.85
C-reactive protein, mg/L	70.07 ± 79.30	78.54 ± 73.28
Prothrombin time (INR)	1.15 ± 0.45	1.21 ± 0.50
**History of comorbidities**		
Chronic kidney disease	93 (3.8)	8 (2.6)
Cancer	1,102 (45.1)	149 (48.1)
Heart failure	56 (2.3)	6 (1.9)
Diabetes	459 (18.8)	65 (21.0)
Cardiac diseases other than heart failure	312 (12.8)	36 (11.6)
Chronic pulmonary disease	169 (6.9)	26 (8.4)

Of the 362 patients with unavailable 30-day follow-up, 52 had missing baseline data and were excluded from this comparison.
